# Correlated neuronal activity and its relationship to coding, dynamics and network architecture

**DOI:** 10.3389/fncom.2014.00102

**Published:** 2014-08-27

**Authors:** Robert Rosenbaum, Tatjana Tchumatchenko, Rubén Moreno-Bote

**Affiliations:** ^1^Department of Applied and Computational Mathematics and Statistics, University of Notre DameNotre Dame, IN, USA; ^2^Center for the Neural Basis of CognitionPittsburgh, PA, USA; ^3^Department Theory of Neural Dynamics, Max Planck Institute for Brain ResearchFrankfurt am Main, Germany; ^4^Research Unit, Parc Sanitari Sant Joan de Déu and Universitat de BarcelonaBarcelona, Spain; ^5^Centro de Investigación Biomédica en Red de Salud Mental (CIBERSAM)Barcelona, Spain

**Keywords:** neuronal correlations, neural synchrony, neural coding, spike train analysis, neuronal networks, noise correlation

Correlated and synchronous activity in populations of neurons has been observed in many brain regions and has been shown to play a crucial role in cortical coding, attention, and network dynamics (Singer and Gray, 1995; Salinas and Sejnowski, 2001). However, we still lack a detailed knowledge of the origin and function, if any, of neuronal correlations. In this Research Topic, new ideas about these long standing questions are put forward. One group of studies in this Research Topic investigates the interaction of neuronal correlations with cellular and circuit mechanisms at the level of single neurons and cell pairs. Bolhasani et al. (2013) study the interaction between direct synaptic coupling between two neurons with correlated stochastic input to the neurons. They find that excitatory synaptic coupling can alter the transfer of pairwise correlations from current input to spike output. Interestingly, there is an optimal value of synaptic coupling strength for which the sensitivity of output correlations to input correlations is maximized.

Bird and Richardson (2014) study the interaction between long term plasticity, synaptic vesicle depletion at multiple release sites and presynaptic spiking correlations. They find that there is an optimal number of release sites for driving postsynaptic spiking when synchrony is present in the presynaptic spike trains. Schwalger and Lindner (2013) investigated correlations between the interspike intervals of oscillator model neurons with adaptation. They reveal a fundamental connection between interval correlations and the phase response curve of the neuron model. They also show that when firing rates are high, negative interval correlations cause long-timescale variability of a model neuron's activity to be small.

A second group of studies in this Research Topic investigates neuronal correlations on the level of networks. The key questions that these studies addressing are: (1) How are pairwise and higher order correlations generated in networks and which of them are important for a given network? and (2) How should we uncover and interpret spike train correlations in a given dataset?

Four studies Zhou et al. (2013), Grytskyy et al. (2013), Barreiro et al. (2014), and Jahnke et al. (2013) have focused on the first question.

Zhou et al. (2013) investigated coupled pairs of neurons receiving temporally correlated input currents. They show that pairs of neurons may be more synchronized if they have some degree of heterogeneity in their intrinsic properties. Temporal correlations in the noise that these neurons receive may also promote synchrony.

Grytskyy et al. (2013) have addressed how recurrent neural networks can support the generation of pairwise correlations. The authors put forward a unified framework for the generation of pairwise correlations in recurrent networks and hypothesize that many different single model neurons, when coupled to a network, may generate the same pairwise correlation structures. Interestingly, the authors could show the equivalence of different single neuron models in a linear approximation to a model with fluctuating continuous variables. This could be a useful tool for assessing correlations across models and experiments.

In a complementary study, Barreiro et al. (2014) have focused on the emergence of pairwise and higher order correlations in retina models. The authors find that maximum entropy pairwise models capture surprisingly well the network spiking dynamics. What is surprising about these results is that higher-order correlations in this type of models can be constrained to be far lower than the statistically possible limits and that their strength depends more on the structure of the common input than on the synaptic connectivity profile.

Jahnke et al. (2013) focused on spike patterns rather than correlations and proposed a mechanism for precise spike time pattern generation and replay in neural networks that lack strong densely connected feed-forward structures. The authors put forward the hypothesis that a non-linearity in synaptic summation rules may explain the lack of observed strong feed-forward structures in live networks.

A team lead by Sonja Grün has tackled the second question, how spike correlations may be detected in a given data set. Torre et al. (2013) have extended our methodical toolbox and proposed a new method for the extraction of statistically overrepresented spike patterns that may be the functionally significant “cell assemblies” proposed by Abeles (1982). The challenge this study has taken on is to extract from large number of simultaneously recorded neurons candidate assemblies that are systematically co-activated. This search algorithm may help to reveal how precise multi-neuron synchronization patterns that go beyond the standard pairwise analysis may relate to behavior.

In an opinion article, Zanin and Papo (2013) also address the second question. They suggest that one has to be cautious about interpreting neuronal correlations between neurons or brain areas, because typical measurements of effective connectivity might lead to false positives even when the neurons or the brain areas are indeed performing independent computations.

A third group of studies in this Research Topic addresses the computational advantages of neuronal correlations in the brain. Kilpatrick (2013) studied neuronal networks that sustain bump attractors, a well-established model for the maintenance of spatial cues in working memory tasks (Funahashi et al., 1989; Wimmer et al., 2014). In these models, the position of the bump undergoes a diffusion process, implying that the encoded memory degrades as the time progresses. Notably, Kilpatrick found that connecting several areas with similar bump attractors resulted in an increased stability of the stored memories because the variability within the areas could be averaged out. However, if the variability across areas was correlated, the diffusion of the bump attractor underwent larger variability. This study, therefore, suggests that correlated noise across neuronal areas can impoverish the precision of the encoding of spatial cues in working memory task.

In another study, Dipoppa and Gutkin (2013) found that correlations might have a positive role in working memory tasks by a mechanism that they named “correlation-induced gating.” These authors and others have previously showed that correlations tend to destabilize the memory trace of an item stored in working memory. This result might suggest that correlations are deleterious for working memory, but Dipoppa and Gutkin argue that this is not the case: correlations in working memory circuits can be strongly beneficial to suppress the harmful interference of distractors, irrelevant items that do not need to be stored in memory to solve the ongoing task. This study, therefore, shows in an elegant way how changing correlations within specific neuronal population can allow for flexible gating of sensory information into working memory circuits.

Previous works have showed that synchronization between neuronal ensembles might play an important role in the binding of features belonging to a same object (Engel and Singer, 2001). In a theoretical work presented in this Research Topic, Finger and Koenig (2014) took an important step forward by showing that binding of features in natural images can be mediated by phase synchronization in a network of neural oscillators. The authors also found that the network, trained with natural images, developed small-world properties, and even allowed binding of features over long distances. This study strongly supports the idea that neuronal correlations in the brain might play an important computational role.

In a study where the LFP and single-cell activity were recorded in the hippocampal formation of epileptic patients, Alvarado-Rojas et al. (2013) found that activity of a sizable fraction of neurons preceded interictal epileptiform discharges, as measured by LFP activity.

These studies give conspicuous examples for the ambivalent nature of neuronal correlations: in some conditions correlations might be a signature of dynamic instability of the network, but in other conditions correlations might be used to perform complex and flexible computations, such as binding or information gating. Although these works have provided new clues about the role of neuronal correlations, there are yet many unsolved questions, such as how neuronal correlations are generated and propagated (Moreno et al., 2002; Moreno-Bote and Parga, 2006; de la Rocha et al., 2007; Ostojic et al., 2009; Renart et al., 2010; Rosenbaum et al., 2010, 2011; Tchumatchenko et al., 2010; Cohen and Kohn, 2011; Tchumatchenko and Wolf, 2011; Helias et al., 2014) and how correlations are shaped by limited information in sensory inputs and by neuronal computations. It is clear that the study of the impact of neuronal correlations on information transmission and brain computation, and vice versa, is still an arena for exciting new discoveries.

## Conflict of interest statement

The authors declare that the research was conducted in the absence of any commercial or financial relationships that could be construed as a potential conflict of interest.
